# Burden of dermatology outpatient and inpatient services in China, 2011–2023

**DOI:** 10.3389/fpubh.2026.1917511

**Published:** 2026-10-02

**Authors:** Jingyi Li, Jiaqi Zeng

**Affiliations:** 1 Department of Dermatology, Huizhou First Hospital, Huizhou, China; 2 Department of Dermatology, Zhuhai People's Hospital (Zhuhai Clinical Medical College of Jinan University), Zhuhai, China

**Keywords:** China, dermatology services, disease burden, regional distribution, temporal trends

## Abstract

**Background:**

This study aimed to describe the temporal trends, regional distribution of dermatology services, and the hospitalization burden of common skin diseases in China (2011–2023) and provide reference for the government about resource allocation.

**Methods:**

Data on dermatology outpatient visits and discharges from public hospitals by age and provinces were sourced from *the China Health Statistics Yearbook.*

**Results:**

From 2011 to 2023, the total number of dermatology outpatient visits and inpatient discharges from public hospitals in China were 1.36 billion and 7.38 million, respectively. Both the number and rate of outpatient and inpatient increased until 2019, declined in 2020, and rebound after 2021. Outpatient visits were highest in the eastern region, whereas inpatients were highest in the western region. The top five provinces for total outpatient visits were Guangdong, Zhejiang, Jiangsu, Shanghai, and Sichuan, for inpatient discharges from public hospitals: Hubei, Sichuan, Shandong, Guangdong, and Henan. Among four skin diseases, eczema/dermatitis had the most hospitalizations (1,209,754), followed by urticaria (719,610), benign skin tumors (653,036), and psoriasis (370,409). Total hospitalization cost for the four diseases was 14.66 billion RMB, with eczema/dermatitis accounting for the largest share (6.23 billion). Psoriasis had the longest hospital stay (median 11.58 days) and highest cost (median 8,880.66 RMB) per patient. Hospitalized for benign skin tumors, psoriasis, eczema/dermatitis, and urticaria were mainly in the 15–44, 15–44, ≥60 and 5–44 age group, respectively.

**Conclusion:**

The burden of skin diseases in China showed an increased trend before the COVID-19 pandemic. Dermatology service in China showed an east–west inverse pattern between outpatient and inpatient care. Resource allocation should consider both outpatient and inpatient data. Eczema/dermatitis had the highest hospitalization burden, while psoriasis had the longest stays and highest costs. Meanwhile, age-stratified strategies should be implemented in skin diseases clinical management.

## Introduction

Skin diseases is one of the most significant health problems on a global scale, significantly affecting patients’ quality of life and imposing a heavy socioeconomic burden (1). The Global Burden of Disease (GBD) study estimated that skin disease is the fourth cause of disability worldwide (2). Lately, studies based on the GBD data have revealed the evolution of skin disease burden. In 2021, the number of new cases of skin diseases worldwide was approximately 4.69 billion, and the resulting years lived with disability ranked seventh among all diseases, equivalent to six in every 10 people having a skin disease (3).

In China, urbanization, demographic shifts, and changes in lifestyle may have altered the burden pattern of skin and subcutaneous diseases (4). During 1990–2021, China experienced a modest increase in the age-standardized incidence of skin, while the age-standardized prevalence rate increased more markedly. Although mortality declined, the overall disease burden did not decrease, and years of life lost remained higher in males (5). The burden varied considerably across different skin diseases. In 2021, psoriasis affected approximately 8.45 million people in China, with the incidence and prevalence rate of 71.17 per 100,000 and 594.14 per 100,000, respectively (6), atopic dermatitis was estimated to affect 15.42 million individuals (7), and there were reported 9.87 million existing cases of urticaria (8).

The burden of skin diseases is an increasing challenge, previous studies have focused on a single disease or specific populations, lacking a systematic evaluation of overall outpatient and inpatient dermatology services. Further, China still lacks nationwide and long-term trend analysis of the dermatological diagnosis and treatment burden. *The China Health Statistics Yearbook*, an annual report compiled and released by National Health Commission of the People’s Republic of China, systematically collects healthcare service data from all provinces and provides a valuable resource for assessing trends in dermatological care. This study analyzed data from *the China Health Statistics Yearbook (2011–2023)* to examine temporal trends and regional distribution of outpatient and inpatient dermatology services, as well as the hospitalization burden of four skin diseases (benign skin tumor, psoriasis, eczema/dermatitis, and urticaria), aiming to inform resource allocation and prevention strategies.

## Materials and methods

### Data source

Data on dermatology outpatient visits, discharges from public hospitals, length of hospital stay, hospitalization costs in China from 2011 to 2023 were obtained from *the China Health Statistics Yearbook* (https://www.nhc.gov.cn/mohwsbwstjxxzx/tjzxtjsj/tjsj_list.shtml), which is compiled and released by National Health Commission of the People’s Republic of China. Subgroup analyses were performed by age group and region. The data cover 31 provinces, autonomous regions, and municipalities in China (excluding Hong Kong, Macao, and Taiwan). In the Yearbook, outpatient visits are recorded by department (dermatology), whereas hospitalization data are available both by department and disease diagnosis categories with corresponding ICD-10 codes. However, individual level diagnostic information is not available in the dataset. Data on four skin diseases (eczema/dermatitis, urticaria, psoriasis, and benign skin tumors) were extracted from *the China Health Statistics Yearbook*. These conditions were selected because these skin diseases completely and consistently reported hospitalization data in the Yearbook over the study period. Provinces were divided into eastern, central, and western regions according to the official classification of China, which is also adopted in *the China Health Statistics Yearbook*. All hospitalization costs were adjusted for inflation to 2023 RMB using the annual healthcare Consumer Price Index (CPI) reported in *the China Health Statistics Yearbook*. The formula was: *adjusted hospitalization cost = original reported hospitalization cost × (CPI_year_ × CPI_year + 1_ ×* … *× CPI_2022_)/100^^(2023-year)^*. The 2023 costs were used as the reference (base year, index = 1.00). Patients were divided into five age groups according to the standard classification of *the China Health Statistics Yearbook*: ≤4 years, 5–14 years, 15–44 years, 45–59 years, and ≥60 years. Hospitalization rate was defined as the number of inpatient discharges due to a skin disease per year divided by the surveyed population in that year. Average outpatient visits (hospitalizations) rate was defined as the total number of outpatient dermatology visits (inpatient discharges) over a given period divided by the total year-end population over the same period. Average hospitalization cost was defined as the total hospitalization cost divided by the total number of hospitalizations during a given period.

As the data were publicly accessible and did not contain any patient-specific information, thereby exempting it from requiring ethical approval for its use.

### Statistical analyses

Data cleaning, hospitalization rate and cost calculation were performed in Microsoft Excel. Statistical analyses were conducted utilizing SPSS version 22.0 (IBM Corp., Armonk, NY, USA), while data visualization was executed using Origin version 2021 (OriginLab Corp., Northampton, MA, USA).

## Results

### Dermatology outpatient and inpatient trends and regional distribution

From 2011 to 2023, the total number of dermatology outpatient visits and inpatient discharges from public hospitals in China were 1.36 billion and 7.38 million, respectively. From 2011 to 2019, both the number and rate of outpatient and inpatient showed an increasing trend, followed by a decline in 2020, and then a rebound after 2021(Figure 1A,B). The annual percentage changes (APC) for the number and rate of outpatient visits and hospitalizations are shown in Table 1.

**Figure 1:**
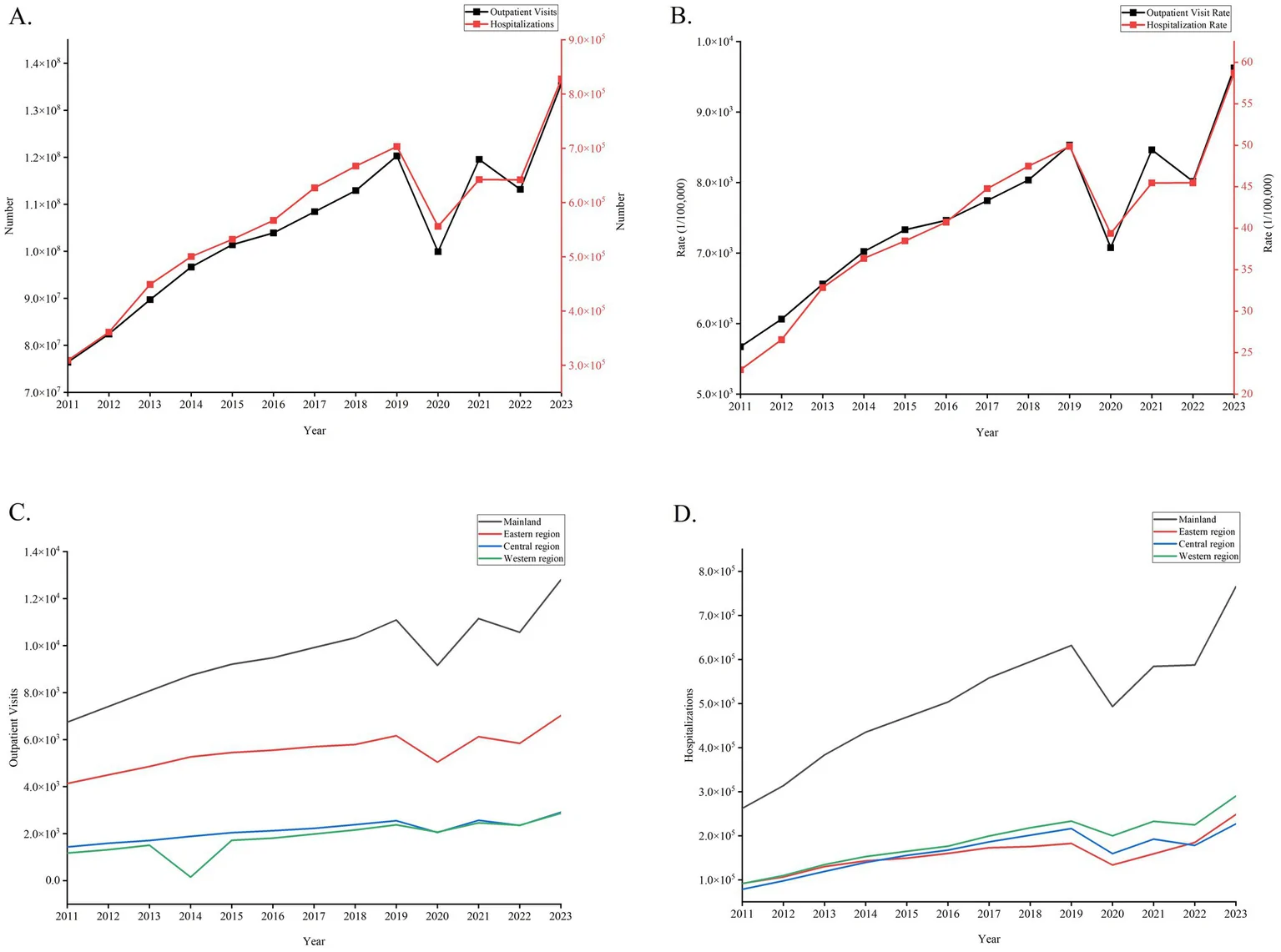
Trends and regional distribution of dermatology outpatient and inpatient service from 2011 to 2023). **(A)** Number of outpatient and hospitalizations; **(B)** outpatient visits rate and hospitalization rate (1/100,000); **(C)** outpatient visits by region; **(D)** hospitalization by region. Rates are crude rates.

**Table 1:** Annual percentage change in overall outpatient visits and hospitalization indicators, 2011–2023.

Year	Outpatient visits (%)	Outpatient visit rate (%)	Hospitalization (%)	Hospitalization rate (%)
2011	–	–	–	–
2012	7.85	6.91	16.86	15.84
2013	8.87	8.23	24.34	23.61
2014	7.73	7.01	11.46	10.71
2015	4.91	4.39	6.36	5.84
2016	2.48	1.81	6.54	5.84
2017	4.34	3.76	10.56	9.94
2018	4.16	3.76	6.43	6.03
2019	6.52	6.17	5.39	5.04
2020	−16.92	−17.04	−20.93	−21.05
2021	19.64	19.60	15.51	15.47
2022	−5.33	−5.27	−0.04	0.02
2023	19.87	20.04	28.92	29.11

In terms of regional distribution, dermatology outpatient visits were highest in eastern region, followed by central region, and lowest in western region. In contrast, inpatient discharges from public hospitals showed an opposite pattern, there were the highest number in western region and the lowest in eastern region (Figure 1C,D). Among the 31 provinces, the top five provinces for total dermatology outpatient visits were Guangdong, Zhejiang, Jiangsu, Shanghai, and Sichuan, for inpatient discharges from public hospitals, the top five were Hubei, Sichuan, Shandong, Guangdong, and Henan (Figure 2). However, the top five for average outpatient visits rate were Shanghai, Beijing, Zhejiang, Jiangsu, and Guangdong, and the top five for average hospitalizations rate were Qinghai, Xinjiang, Hubei, Sichuan, and Yunnan (Figure 3).

**Figure 2:**
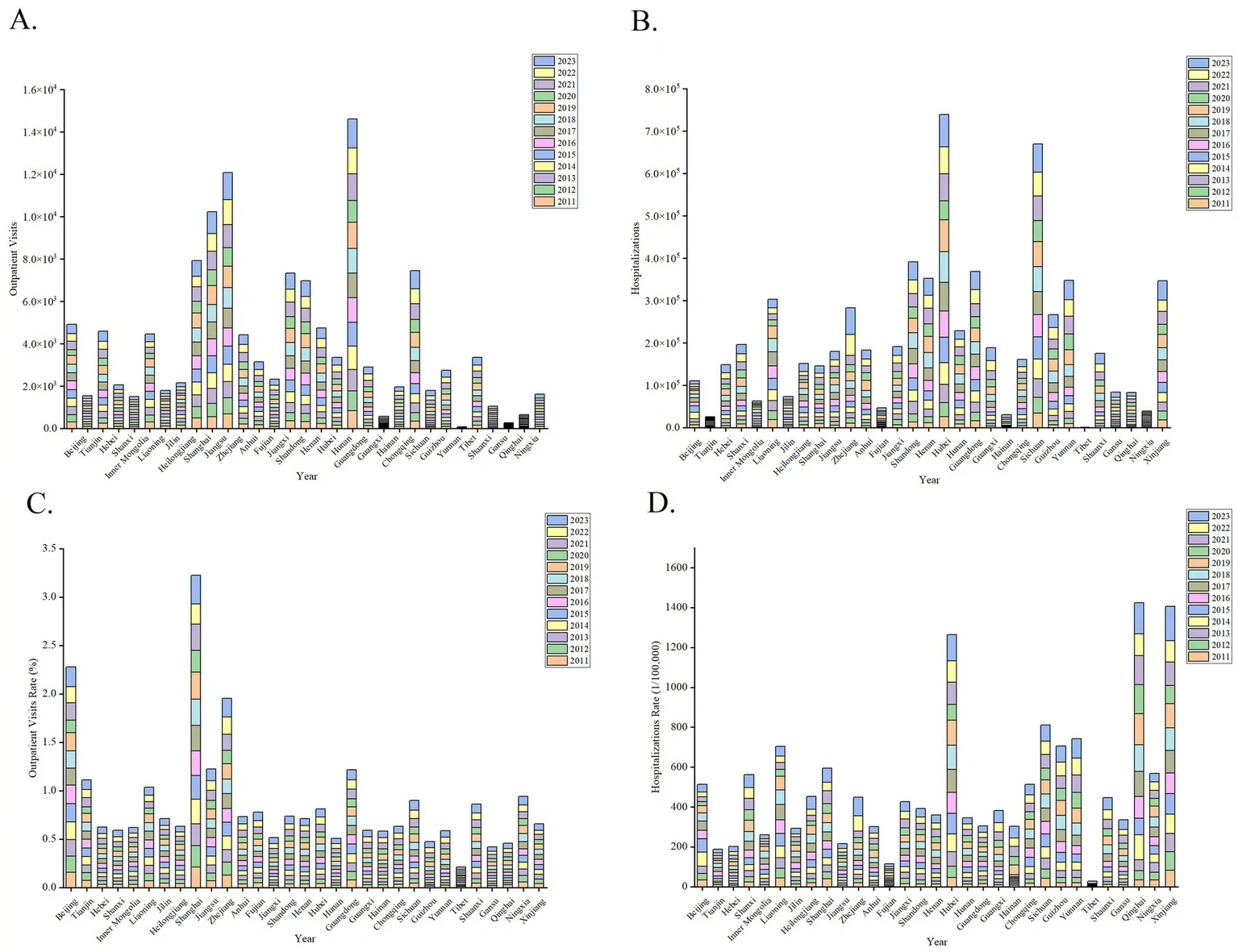
Provincial distribution of dermatology outpatient visits and inpatient discharges from 2011 to 2023. **(A)** Number of outpatient visits; **(B)** number of hospitalizations; **(C)** outpatient visits rate (%); **(D)** hospitalization rate (1/100,000). Rates are crude rates.

**Figure 3:**
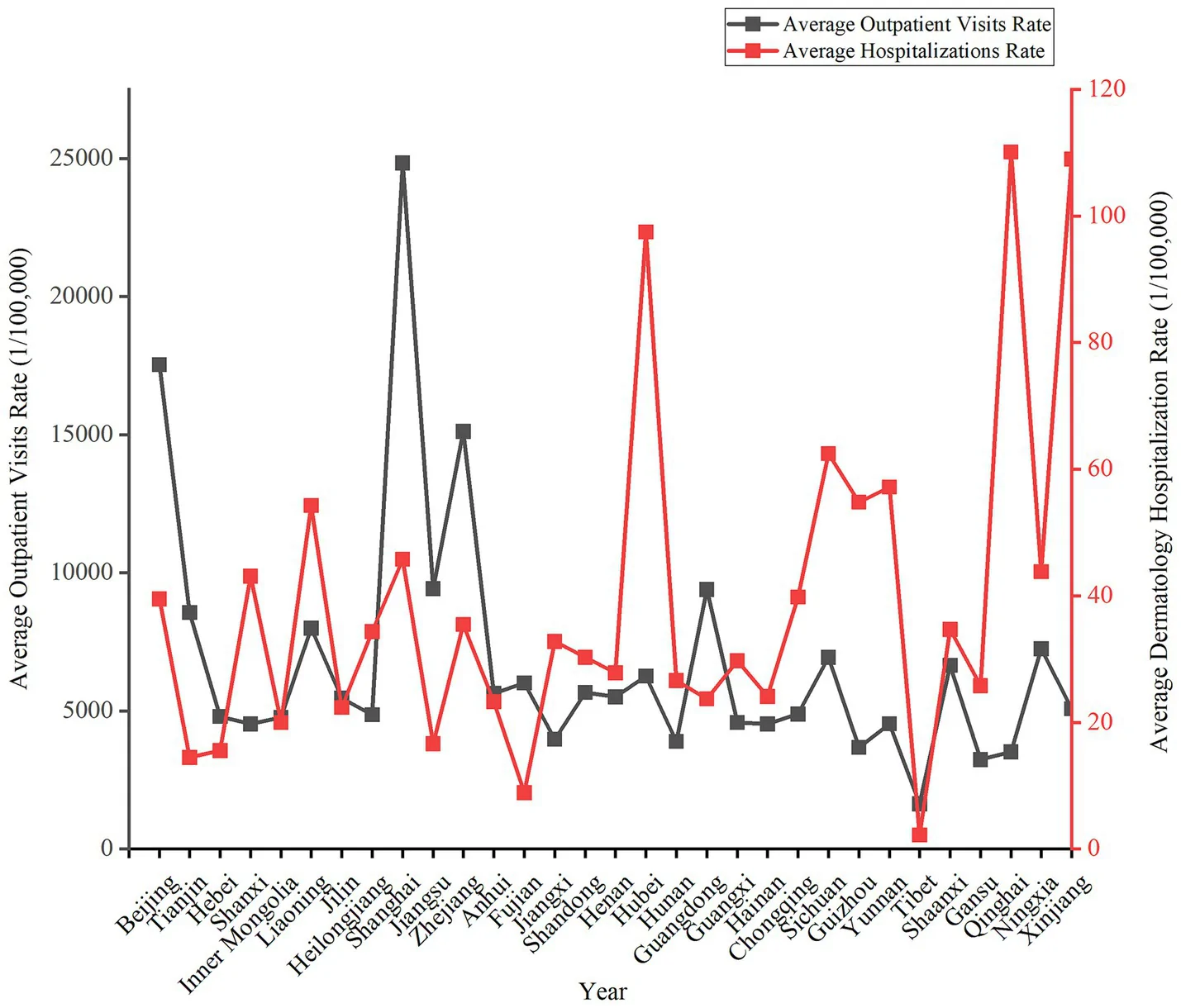
Provincial distribution of average outpatient rate and average hospitalizations rate (1/100,000) from 2011 to 2023. Rates are crude rates.

### Hospitalization burden of the four skin diseases

From 2011 to 2019, the number of hospitalizations and hospitalization rates for benign skin tumors, eczema/dermatitis, psoriasis, and urticaria showed increasing trends, declined in 2020, and rebound in 2021. Eczema/dermatitis had the highest total number of hospitalizations (1,209,754), followed by urticaria (719,610), benign skin tumors (653,036), and psoriasis (370,409). The average hospitalization rates followed the same order and were 6.6883/100,000, 3.9785/100,000, 3.6104/100,000, and 2.0479/100,000, respectively (Figure 4A,B). The APC for the number and rate of hospitalizations for above four diseases are shown in Table 2.

**Figure 4:**
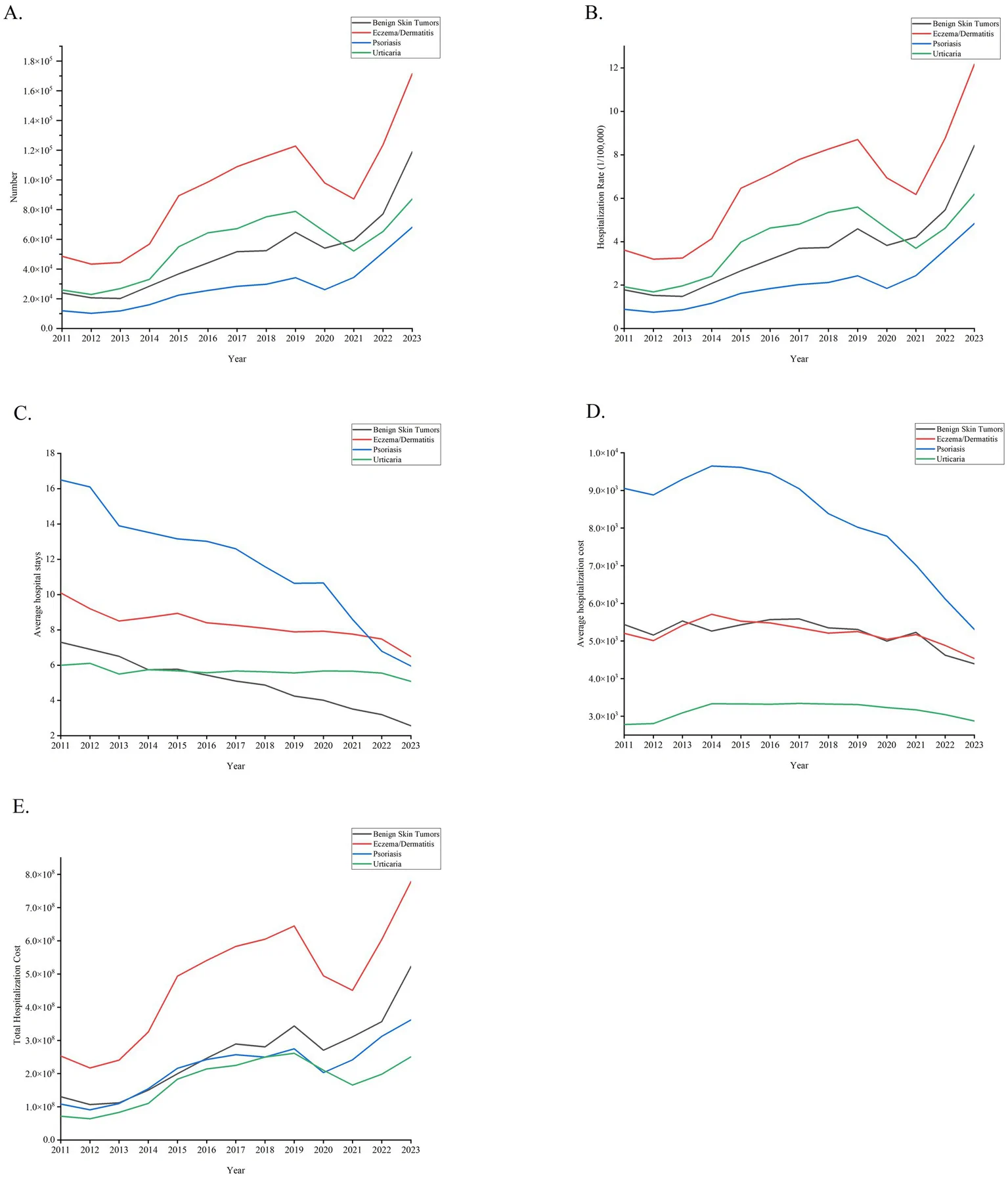
Hospitalization burden of four skin diseases (benign skin tumors, psoriasis, eczema/dermatitis, and urticaria) from 2011 to 2023. **(A)** Number of hospitalizations; **(B)** hospitalization rate (1/100,000); **(C)** average length of hospital stays (days); **(D)** average hospitalization cost (RMB); **(E)** total hospitalization cost (RMB). Rate is crude rate and all costs are adjusted to 2023 RMB using healthcare CPI.

**Table 2:** Annual percentage change in hospitalization indicators for four skin diseases, 2011–2023.

Year	Hospitalization (%)				Hospitalization rate (%)			
	Benign skin tumors	Eczema /dermatitis	Psoriasis	Urticaria	Benign skin tumors	Eczema /dermatitis	Psoriasis	Urticaria
2011	–				–			
2012	−13.69	−10.82	−14.21	−11.64	−14.44	−11.60	−14.96	−12.41
2013	−2.11	2.42	15.26	17.45	−2.69	1.82	14.58	16.76
2014	40.92	28.19	35.48	23.21	39.98	27.33	34.58	22.39
2015	28.68	56.84	40.35	66.44	28.05	56.07	39.66	65.63
2016	20.37	10.46	14.15	16.98	19.59	9.74	13.41	16.22
2017	16.89	10.46	10.74	4.31	16.24	9.84	10.12	3.73
2018	1.36	6.51	4.94	11.82	0.97	6.11	5.54	11.39
2019	23.45	5.76	14.97	4.89	23.04	5.41	14.59	4.54
2020	−16.43	−20.17	−23.79	−17.41	−16.55	−20.29	−23.90	−17.53
2021	9.95	−11.05	31.90	−19.90	9.91	−11.08	31.86	−19.93
2022	29.67	41.87	48.35	25.01	29.75	41.95	48.44	25.08
2023	54.21	38.72	33.49	33.71	54.44	38.92	33.69	33.91

From 2011 to 2023, the average length of hospital stays for above four diseases showed decreased trends. Psoriasis had the longest hospital stay, with a median average length of hospital stay of 11.58 days (range: 5.95–16.50 days), and had the highest median average hospitalization cost of 8,880.66 RMB (decreasing from 9,649.53 RMB to 5,306.94 RMB). For the other three diseases, the median average length of hospital stay and median average hospitalization cost were as follows: benign skin tumors: 5.10 days (2.56–7.30 days) and 5,306.74 RMB (4,394.66–5588.03 RMB); eczema/dermatitis: 8.26 days (6.48–10.10 days) and 5,207.52 RMB (4,535.00-5,711.48 RMB); urticaria: 5.66 days (5.08–6.10 days) and 3,229.15 RMB (2,779.62-3,342.65 RMB; Figure 4C,D).

During this period, the estimated total hospitalization cost for the above four skin diseases in public hospitals was 14,661.63 million RMB. Eczema/dermatitis had the highest cost at 6,230.69 million RMB, followed by benign skin tumors (3,319.20 million RMB), psoriasis (2,822.75 million RMB), and urticaria (2,288.99 million RMB; Figure 4E).

### Hospitalization age distribution of the four skin diseases

From 2011 to 2023, patients hospitalized for benign skin tumors and psoriasis were mainly in the 15–44 age group; eczema/dermatitis peaked in those aged ≥60 years; and urticaria was most frequent in the 5–44 age group (Figure 5).

**Figure 5:**
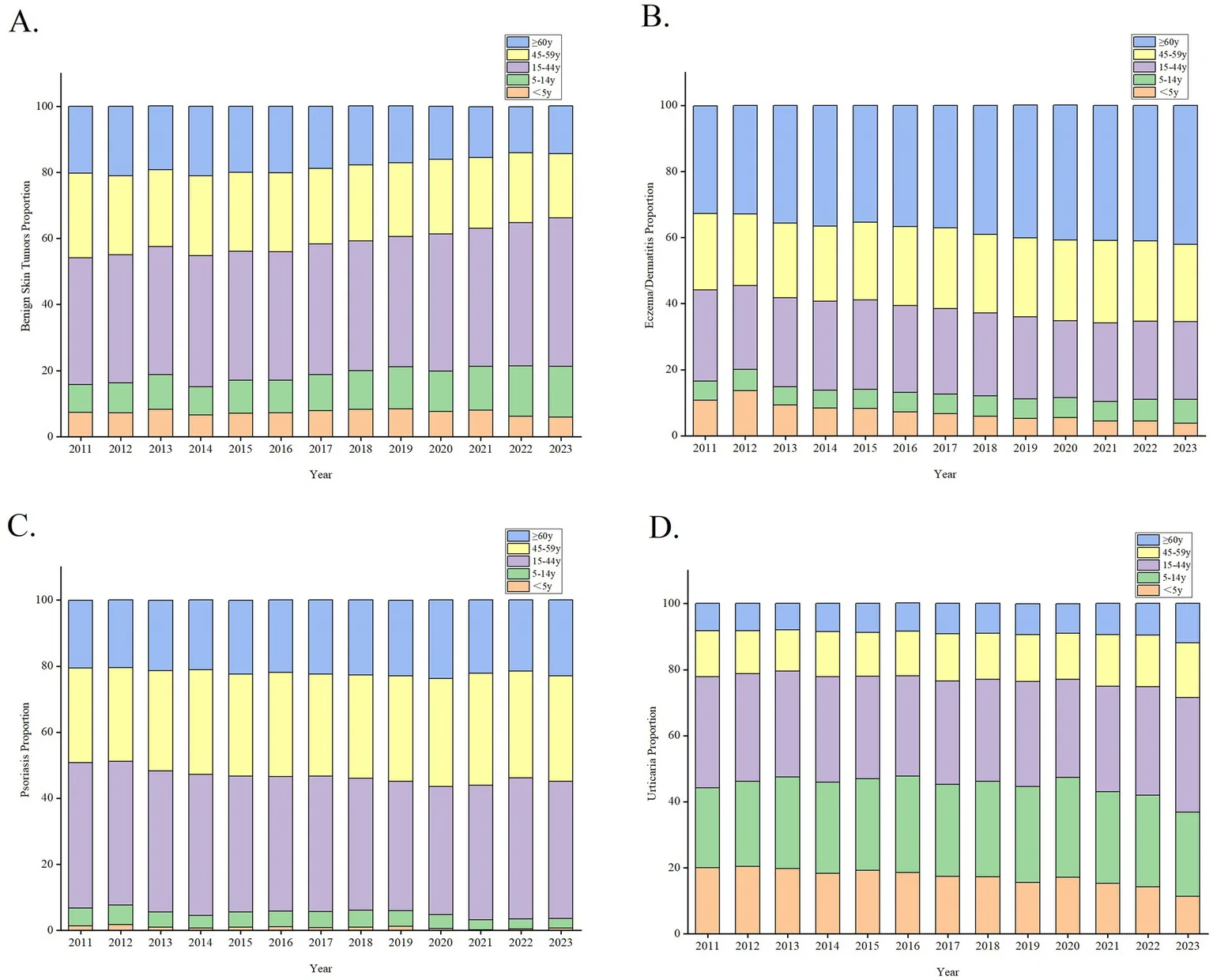
Age distribution of hospitalized patients with four skin diseases in China from 2011 to 2023. **(A)** Benign skin tumors; **(B)** eczema/dermatitis; **(C)** psoriasis; **(D)** urticaria. Age groups: ≤4, 5–14, 15–44, 45–59, and ≥60 years.

## Discussion

In this study, we had analyzed temporal trends and regional distribution of outpatient and inpatient dermatology services, as well as the hospitalization burden of four common skin diseases (benign skin tumors, psoriasis, eczema/dermatitis, and chronic urticaria), based on the data of *the China Health Statistics Yearbook (2011–2023)*. We found that the steady increase in outpatient and inpatient dermatology visits from 2011 to 2019, which is consistent with the improvement in healthcare accessibility in China (9). However, a decline in dermatology visits was observed in 2020, coinciding with the COVID-19 pandemic. During this period, non-urgent medical services were reduced in China, and patients delayed seeking care (10, 11). On January 24, 2020, West China Hospital, Sichuan University temporarily delayed the elective surgeries, and retained only emergency surgeries; the number of dermatology outpatient clinics was reduced from 8 to 4 (11). Approximately 32% of Chinese adults delayed or avoided medical seeking care during the COVID-19 epidemic (10). The rebound after 2021 indicates that dermatology care gradually recovered, which is consistent with reports from other countries (12). In addition, this period also coincided with the implementation of healthcare system reforms and the expansion of health insurance coverage in China to improve healthcare accessibility and utilization (13).

We found that dermatology outpatient visits were highest in the eastern region and lowest in the western region, whereas inpatient in dermatology showed an opposite pattern. However, these regional comparisons should be interpreted with caution, as regional differences in population size, healthcare resources, and service accessibility may also influence the observed patterns. In 2021, the per capita GDP of eastern China was 1.68 times that of western China (14). People living in the eastern developed areas are more likely to use outpatient care, while those in western underdeveloped areas are more likely to use inpatient care (15). In eastern region, higher economic levels attract more medical resources and professionals through better salaries and research opportunities, and higher-income groups have a higher rate of outpatient service utilization. In western region, limited primary care capacity and lower healthcare accessibility lead to more hospitalization (16). Previous studies have shown that the density of healthcare infrastructure and healthcare workforce in eastern region is higher than that in western region (17). The physician distribution levels in eastern provinces such as Beijing, Zhejiang, Shanghai, Jiangsu, Shandong, and Tianjin are higher than the national level (18). Using only outpatient or inpatient indicators alone may obscure the true regional distribution of disease burden, these data should be considered together in health planning and resource allocation.

Eczema/dermatitis exhibited highest number and rate of hospitalization among the four diseases, consistent with its high prevalence, chronic and relapsing characteristics and major impact on quality of life. Atopic dermatitis affects approximately 204 million individuals worldwide, exerting a significant psychosocial impact on patients and their families (19). Notably, psoriasis had the longest average hospital stay (median 11.58 days, range 5.95–16.50 days) and highest average hospitalization cost (median 8,880.66 RMB) among the four diseases, while average hospital stays for these diseases have been declining. The overall downward trend in average length of hospital stay reflects improved healthcare efficiency and the impact of payment system reforms, such as diagnosis-related groups (DRG). It was found that DRG reform shortened the average length of hospital stay by 2 days (20). The longer hospital stay is explained by psoriasis subtypes and respiratory tract infections, pustular psoriasis and erythrodermic psoriasis were significantly associated with an increased hospital stay (21). A retrospective study of 322 patients with moderate-to-severe pustular psoriasis reported that biologic use increased from 0 to 57.0%, and biologic monotherapy was associated with shorter hospital stays and faster pustule clear than conventional therapy (22). However, biologics were the primary driver of healthcare costs in psoriasis, with biologics users incurring more than double the direct medical cost of non-biologic users (23). From 2011 to 2023, the total hospitalization cost for the aforementioned four skin diseases in Chinese public hospitals was estimated at 14.66 billion RMB (eczema/dermatitis: 6.23 billion; benign skin tumors: 3.32 billion; psoriasis: 2.82 billion; urticaria: 2.29 billion), reflecting a substantial economic burden. Notably, the hospitalization costs in this study were adjusted for inflation to 2023 RMB using the healthcare CPI, but were not adjusted for regional differences in purchasing power.

In our study, benign skin tumors and psoriasis were prominent in the 15–44 age group. This pattern is consistent with the peak age at psoriasis onset is 20–29 years in Asia (24). However, according to GBD 2021 data, the burden of psoriasis is prominent in the older adults, and its incidence is positively correlated with economic development (25). However, we found that the proportion of eczema/dermatitis patients was highest in the ≥60 years age group. Aging is associated with epidermal barrier dysfunction, decreased stratum corneum hydration, and increased skin surface pH, coinciding with a higher risk of eczema/dermatitis in older adults (26). In contrast, urticaria was most common in the 5–44 age group. However, atopic constitution is relatively common in childhood (27). A retrospective study on pediatric chronic urticaria conducted at Shanghai Children’s Hospital reported 211 cases (30.2%) with atopic comorbidities, with a median age of onset of 4.0 (2.0–7.0) years (27). Urticaria remains common in the 20–44 age group. Previous studies report that over half of chronic urticaria cases involve an autoimmune mechanism, and the disease is more common in patients >20 years (28). Moreover, T cells are more responsive to antigens in young adults, and Th17/IL-17 pathway activation are observed in persistent chronic urticaria (29). Thus, age-stratified strategies should be implemented in skin diseases clinical management.

This study has several limitations. First, the data are obtained from the *China Health Statistics Yearbook* without individual level information, we were unable to analyze influencing factors such as disease severity, comorbidities, and specific treatment. Second, the lack of outpatient costs, out-of-pocket expenses, non-public hospital data, and detailed cost components (e.g., drugs, procedures, bed fees) may underestimate the total economic burden. Third, we did not decompose inpatient costs, nor could we evaluate the impact of insurance policies on hospitalization expenses. Fourth, the regional comparisons may be confounded by other factors, such as regional differences in population structure, healthcare resources, and service accessibility. Although we used crude rates to adjust for population size, residual confounding remains possible, and the observed patterns may not fully accurately reflect the true regional differences in disease burden. Fifth, the rates presented are crude ratios and have not been age-standardized. Age-standardized rates could not be calculated due to the aggregated nature of the Yearbook data. Sixth, the age groups are broad, thus may mask certain intergroup variations, but finer stratification was not possible due to the limitation of the Yearbook data.

## Conclusion

From 2011 to 2023, the burden of skin diseases in China showed an increased trend before the COVID-19 pandemic, and there were several regional and age differences. Dermatology outpatient visits were highest in the eastern region, whereas inpatients were highest in the western region. Thus, resource allocation should consider both outpatient and inpatient metrics simultaneously rather than relying on a single indicator. However, these regional comparisons should be interpreted with caution, because regional differences in population structure, healthcare resources, and service accessibility may influence the observed patterns. Hospitalized for benign skin tumors, psoriasis, eczema/dermatitis, and urticaria were mainly in the 15–44, 15–44, ≥60 and 5–44 age group, respectively. Age-stratified strategies should be implemented in skin diseases clinical management, including early detection and standardized treatment for young adults, skin barrier maintenance and comorbidity management for the older adults, and atopy-focused strategies for urticaria. Eczema/dermatitis was the leading cause of hospitalization, while psoriasis had the longest hospital stay and the highest average hospitalization costs, highlighting the need for ongoing surveillance of treatment patterns and their economic impact.

## Data Availability

Publicly available datasets were analyzed in this study. Data on dermatology outpatient visits, discharges from public hospitals, length of hospital stay, hospitalization costs in China from 2011 to 2023 were obtained from *the China Health Statistics Yearbook* (https://www.nhc.gov.cn/mohwsbwstjxxzx/tjzxtjsj/tjsj_list.shtml).
